# Mechanical complications and outcomes following invasive emergency procedures in severely injured trauma patients

**DOI:** 10.1038/s41598-018-22457-9

**Published:** 2018-03-05

**Authors:** Manuel F. Struck, Johannes K. M. Fakler, Michael Bernhard, Thilo Busch, Patrick Stumpp, Gunther Hempel, André Beilicke, Sebastian N. Stehr, Christoph Josten, Hermann Wrigge

**Affiliations:** 1 Department of Anesthesiology and Intensive Care Medicine, University Hospital Leipzig, Liebigstr. 20, 04103 Leipzig, Germany; 2 Department of Orthopedics, Trauma and Plastic Surgery, University Hospital Leipzig, Liebigstr. 20, 04103 Leipzig, Germany; 3 Emergency Department, University Hospital Leipzig, Liebigstr. 20, 04103 Leipzig, Germany; 4 Department of Diagnostic and Interventional Radiology, University Hospital Leipzig, Liebigstr. 20, 04103 Leipzig, Germany

## Abstract

This study aimes to determine the complication rates, possible risk factors and outcomes of emergency procedures performed during resuscitation of severely injured patients. The medical records of patients with an injury severity score (ISS) >15 admitted to the University Hospital Leipzig from 2010 to 2015 were reviewed. Within the first 24 hours of treatment, 526 patients had an overall mechanical complication rate of 26.2%. Multivariate analysis revealed out-of-hospital airway management (OR 3.140; 95% CI 1.963–5.023; p < 0.001) and ISS (per ISS point: OR 1.024; 95% CI 1.003–1.045; p = 0.027) as independent predictors of any mechanical complications. Airway management complications (13.2%) and central venous catheter complications (11.4%) were associated with ISS >32.5 (p < 0.001) and ISS >33.5 (p = 0.005), respectively. Chest tube complications (15.8%) were associated with out-of-hospital insertion (p = 0.002) and out-of-hospital tracheal intubation (p = 0.033). Arterial line complications (9.4%) were associated with admission serum lactate >4.95 mmol/L (p = 0.001) and base excess <−4.05 mmol/L (p = 0.008). In multivariate analysis, complications were associated with an increased length of stay in the intensive care unit (p = 0.019) but not with 24 hour mortality (p = 0.930). Increasing injury severity may contribute to higher complexity of the individual emergency treatment and is thus associated with higher mechanical complication rates providing potential for further harm.

## Introduction

The resuscitation of severely injured patients requires invasive emergency procedures to treat life-threatening conditions. These procedures include airway management, chest tube insertion, central venous catheterization (CVC), and arterial line placement and require profound professional experience for a safe and fast performance. Any procedure-related complication may delay lifesaving diagnostic and therapeutic measures and should be avoided^1-3^.

Data on the process quality of invasive emergency procedures in severely injured patients are available for either out-of-hospital^4–7^ or resuscitation room settings^8–10^, whereas studies elucidating the entire acute care phase including the first 24 hours after injury are scarce^11^. This time period is crucial in the management of severely injured patients since absence, delay, or failure of life-saving procedures may effect patient outcome^12^. Many studies have shown that that early causes of death in the acute care phase are associated with emergency measures^13^. Thus, investigating mechanical complications of emergency procedures might help to change practice patterns in order to reduce preventable deaths, which are reported ranging from 12.3 to 58% in trauma patients^14–17^. In one study with severe trauma patients, 56% of endotracheal tubes, chest tubes and CVCs were documented by postmortem computed tomography (CT) to be misplaced^18^. To which extent mechanical complications of inserted devices contribute to outcomes is unknown.

The aim of this study was to explore the incidence of mechanical complications of airway management (including tracheal intubation, supraglottic airway device placement and bag valve mask ventilation), chest tube insertion and invasive vascular access with respect to the possible effects on outcome beginning from treatment by a physician staffed out-of-hospital emergency medical service (EMS), to the resuscitation room, to the operating room, and to the initial intensive care unit (ICU) stay until the first 24 hours after hospital admission. Mechanical complications were classified as multiple intubation attempts; unsuccessful tracheal intubation requiring supraglottic airway devices or bag valve mask ventilation for rescue management, undetected esophageal and bronchial intubation at resuscitation room admission, chest tube malfunction; vascular access with multiple puncture attempts, accidental arterial puncture, or associated hematoma and/or pneumothorax, CVC and arterial line malpositions, and loss of guidewires.

We hypothesized that complication rates would be associated with different operator-independent factors including intervention environment, time of day, patient condition, and pre-injury morbidity.

## Results

### Patients’ characteristics with and without mechanical complications

**Figure 1:**
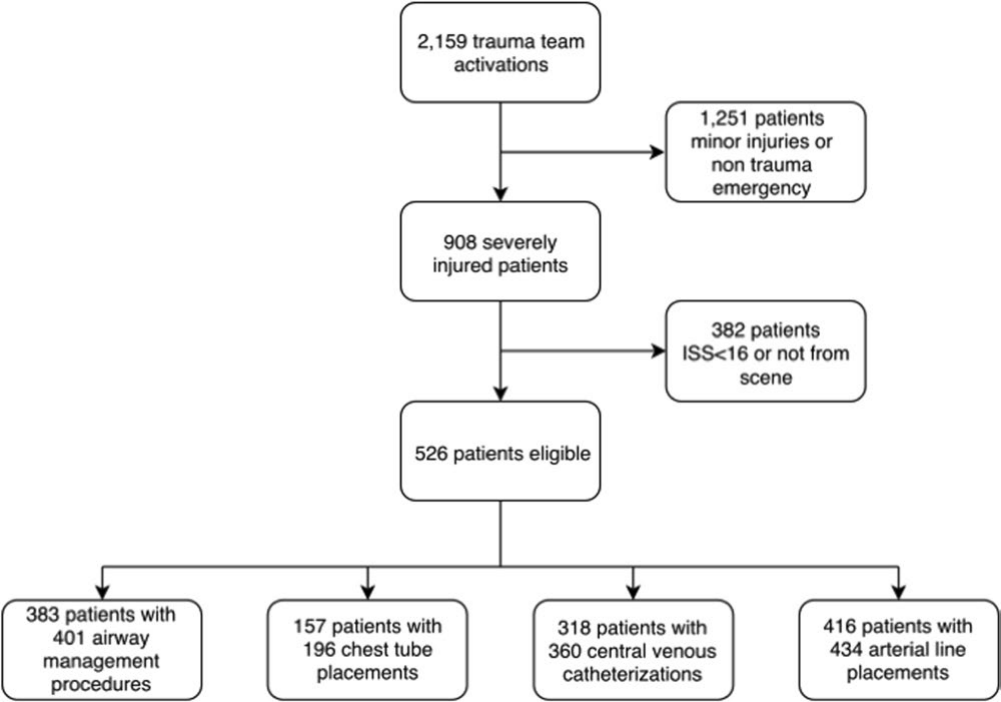
Study flow chart. ISS; injury severity score.

**Figure 2:**
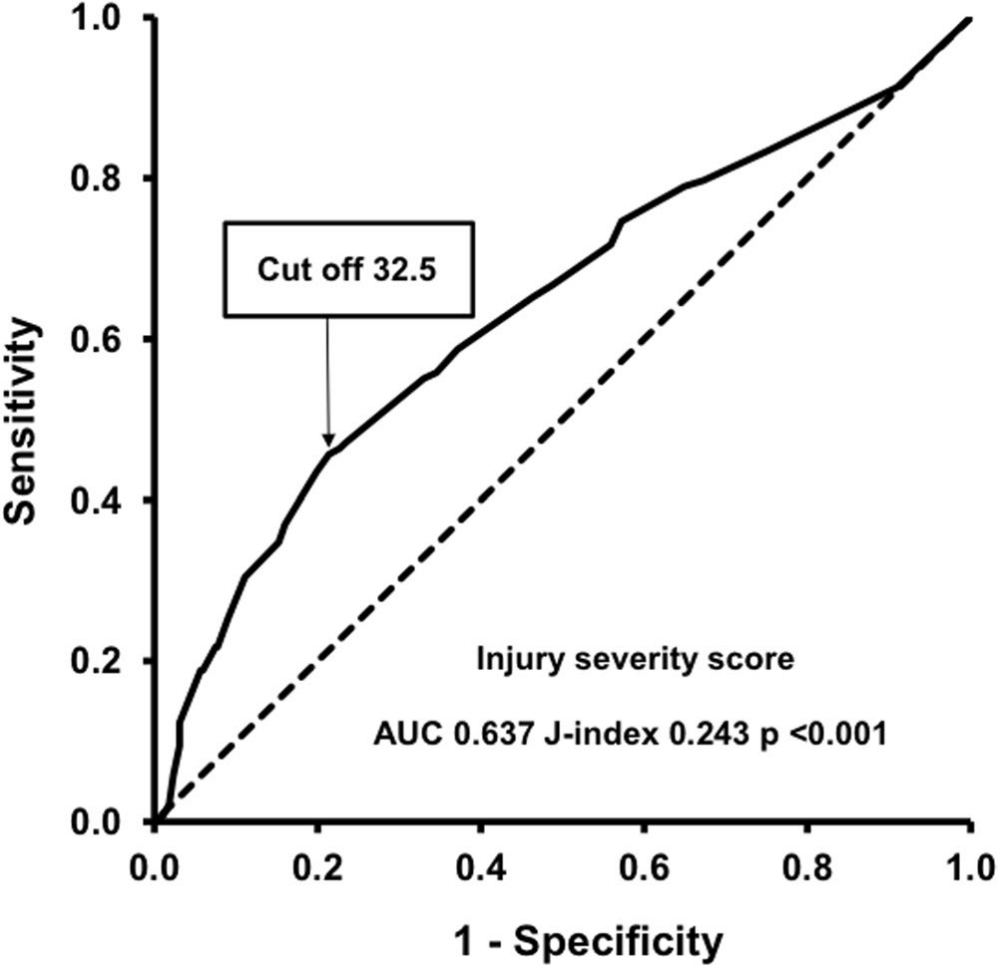
Injury severity score relation with mechanical complications of invasive emergency procedures.

Five hundred and twenty-six patients fit the eligibility criteria and were included (Fig. 1). The mean age was 51 ± 22 years and 372 (70.7%) were males. Injuries were caused by traffic accidents (54.7%), falls (31.9%) and other trauma mechanisms (13.4%). Further demographic data and characteristics are presented in Table 1. Among all patients, 138 (26.2%) suffered at least one mechanical complication related to emergency procedures (Table 2). Of these, 110 patients (79.7%) had only one complication, 25 patients (18.1%) had two complications (n = 9 for airway management and CVC placement, n = 7 for chest tube and CVC placement, n = 6 for CVC placement and arterial line placement, and n = 3 for four other combinations) and five patients (3.6%) had three combinations of complications. Multivariate analysis revealed out-of-hospital airway management (OR 3.140; 95% CI 1.963–5.023; p < 0.001) and injury severity score (ISS) (per ISS point: OR 1.024; 95% CI 1.003–1.045; p = 0.027) as independent predictors of any mechanical complications (Table 3). Receiver operating characteristic (ROC) curve analysis of ISS revealed and area under the curve (AUC) 0.637 (p < 0.001), sensitivity and specificity of 0.457 and 0.786, respectively (cutoff 32.5; J-index 0.243) (Fig. 2). Further stepwise logistic regression analysis (including ISS >32.5, out-of-hospital airway management, admission body temperature, admission hemoglobin, admission base excess, admission serum lactate, red blood cell transfusion <24 hours, noradrenaline dosage <24 hours) confirmed out-of-hospital airway management (OR 3.327; 95% CI 2.1 48–5.152; p < 0.001) and ISS cutoff above 32.5 points (OR 2.119; 95% CI 1.359–3.306; p = 0.001) as predictors of any mechanical complications with a sensitivity of 36.6% and a specificity of 87.7%.

**Table 1:** Characteristics of severely injured patients with and without mechanical complications of invasive emergency procedures.

	Total (n = 526)	Any complication <24 h (n = 138)	No complication <24 h (n = 388)	Univariate p value
Age, years; mean (SD)	51 (22)	49 (20)	51 (22)	0.248
Male; n (%)	372 (70.7)	99 (71.7)	273 (70.4)	0.760
BMI; mean (SD)	25.8 (4.1)	26.0 (4.3)	25.7 (4.0)	0.390
ASA class >2; n (%)	85 (16.2)	18 (13.0)	67 (17.3)	0.247
Pre-injury anticoagulation; n (%)	74 (14.1)	16 (11.6)	58 (14.9)	0.330
Admission daytime 8am–7pm; n (%)	372 (70.1)	96 (69.6)	272 (70.3)	0.874
ISS; mean (SD)	27.8 (12.4)	32.7 (14.2)	26.1 (11.2)	<*0.001*
AIS head >3; n (%)	166 (31.6)	46 (33.3)	120 (30.9)	0.602
Out-of-hospital airway management; n (%)	239 (45.4)	97 (70.3)	142 (36.6)	<*0.001*
Admission SBP, mmHg; mean (SD)	120 (36)	110 (40)	123 (34)	*0.001*
Admission temperature <35.2 °C; n (%)	35.4 (1.5)	35.2 (1.6)	35.6 (1.4)	*0.012*
Admission Hb, mmol/L; mean (SD)	7.47 (1.54)	7.13 (1.62)	7.59 (1.49)	*0.004*
Admission BE, mmol/L; mean (SD)	−2.77 (5.25)	−4.38 (5.61)	−2.20 (5.01)	<*0.001*
Admission Lactate, mmol/L; mean (SD)	3.1 (3.2)	3.8 (3.7)	2.9 (2.9)	*0.006*
Red blood cells <24 h; mean (SD)	2.8 (7.8)	4.8 (11.4)	2.1 (5.7)	*0.008*
Noradrenaline <24 h, μg/kg/min; mean (SD)	0.23 (0.42)	0.37 (0.52)	0.18 (0.37)	<*0.001*
Time to surgery <24 h, h; mean (SD)	1.54 (3.57)	1.36 (2.80)	1.61 (3.81)	0.475
Ventilator days; mean (SD)	4.9 (8.4)	7.7 (10.6)	3.8 (7.3)	<*0.001*
ICU LOS, days; mean (SD)	10.4 (13.4)	14.2 (15.3)	9.1 (12.4)	<*0.001*
24 h mortality; n (%)	41 (7.8)	16 (11.6)	25 (6.4)	0.053
30 day mortality; n (%)	86 (16.3)	29 (21.0)	57 (14.7)	0.084

CI; confidence interval, SD; standard deviation, BMI; body mass index, ISS; injury severity score, ASA; American Society of Anesthesiologists, AIS; abbreviated injury severity. SBP; systolic blood pressure, Hb; hemoglobin, BE; base excess, ICU LOS; intensive care unit length of stay; italicized p values indicate statistical significance (p < 0.05).

**Table 2:** Incidence of mechanical complications of invasive emergency procedures related to performance environments.

Procedure	Total	EMS	Resuscitation room	Operating room	ICU
Airway management; n (%)	53/401 (13.2)	40/239 (16.7)	9/67 (13.4)	0/74 (0)	4/21 (19.0)
Chest tube placement; n (%)	31/196 (15.8)	8/35 (22.8)	16/88 (18.2)	3/26 (11.5)	4/47 (8.5)
CVC placement; n (%)	41/360 (11.4)	1/3 (33.3)	24/191 (12.5)	3/47 (6.4)	13/119 (10.9)
Arterial line placement; n (%)	41/434 (9.4)	0/0 (0)	32/291 (11.0)	0/54 (0)	9/89 (10.1)

CVC; central venous catheter, EMS; emergency medical service, ICU; intensive care unit.

**Table 3:** Multivariate analysis of mechanical complications.

Parameter	Regression coefficient	Standard error	Odds radio	95% CI lower value	95% CI upper value	Multivariate p value
ISS	0.023	0.011	1.024	1.003	1.045	*0.027*
Out-of-hospital airway management	1.144	0.240	3.140	1.963	5.023	*0.000*
Admission SBP (mmHg)	0.003	0.004	1.003	0.995s	1.010	0.485
Admission temperature (°C)	0.002	0.086	1.002	0.847	1.186	0.978
Admission Hb (mmol/L)	0.034	0.085	1.035	0.876	1.222	0.689
Admission base excess (mmol/L)	−0.048	0.034	0.953	0.892	1.020	0.164
Admission Lactate (mmol/L)	−0.072	0.054	0.931	0.836	1.035	0.186
RBC/24 h (n)	0.013	0.017	1.013	0.980	1.046	0.444
Noradrenaline/24 h (µg/kg/min)	0.259	0.314	1.296	0.700	2.400	0.410
Constant	−3.007	3.077				0.329

ISS; injury severity score, SBP; systolic blood pressure, Hb; hemoglobin, RBC; red blood cell transfusion, CI; confidence interval.

Logistic Regression including n = 383 patients without complications and n = 134 patients with complications;

Hosmer-Lemeshow goodness-of-fit test: p = 0.629; Nagelkerke’s R^2^: 0.156; model chi-square: 58.2, p < 0.001. Sensitivity 16.4% (22/134) correctly predicted patients with complications, specificity 97.1% (372/383) correctly predicted patients without complications; italicized p values indicate statistical significance (p < 0.05).

In univariate analysis, ventilator days and ICU length of stay (LOS) of patients with any complication were significantly increased compared to patients without complications (7.7 vs. 3.8 days, p < 0.001 and 14.2 vs. 9.1 days, p < 0.001, respectively) (Table 1). The association of any mechanical complication with ICU LOS was confirmed in robust multiple linear regression analysis (p = 0.019) (Table 4). ICU LOS was further associated with admission serum lactate (p = 0.032), AIS head >2 (p = 0.005) and out-of-hospital airway management (0.027). The 24-hour mortality and 30-day mortality tended to be higher but remained below statistical significance (11.6 vs. 6.4%, p = 0.053 and 21.0 vs. 14.7%, p = 0.084, respectively). Logistic regression analysis of the 24 hour and 30 day mortality did not identify the incidence of mechanical complications as significant predictors (Tables 5 and 6).

**Table 4:** Predictors of intensive care unit length of stay.

Parameter	Regression coefficient	Standard error	95% CI Lower value	95% CI Upper value	Multivariate p value	Univariate p value
Constant	2.682	1.626	−0.667	5.740	0.112	
ISS	0.198	0.069	0.062	0.353	0.006	<*0.001*
Admission Base excess (mmol/L)	−0.126	0.218	−0.547	0.302	0.582	*0.013*
Admission Lactate (mmol/L)	−0.677	0.311	−1.293	−0.074	*0.032*	*0.017*
RBC/24 h (n)	0.136	0.153	−0.070	0.535	0.305	0.058
Noradrenaline (µg/kg/min)	−3.469	2.404	−8.048	1.332	0.123	<*0.001*
Time to surgery (hours)	0.083	0.111	−0.119	0.318	0.459	<*0.001*
ASA 3	0.817	1.914	−3.013	4.723	0.651	0.053
Anticoagulation	3.851	2.027	−0.163	7.810	0.064	*0.003*
AIS head >3	4.195	1.492	1.277	7.182	*0.005*	*0.002*
Out-of-hospital airway management	3.611	1.551	0.580	6.847	*0.027*	*0.001*
Total incidence of complications	3.495	1.508	0.553	6.438	*0.019*	<*0.001*

ISS; injury severity score, RBC; red blood cell transfusion, AIS; abbreviated injury severity, CI; confidence interval for regression coefficient. Robust multiple linear regression including data from n = 514 patients (R = 0.357, p < 0.001; Durbin Watson statistics 2.008). Confidence intervals and standard errors based on 1000 bootstrap samples; italicized p values indicate statistical significance (p < 0.05).

**Table 5:** Predictors of 24-hour mortality.

Parameter	Regression coefficient	Standard error	Odds radio	95% CI lower value	95% CI upper value	Multivariate p value	Univariate p value
Constant	5.407	6.295	222.975			0.390	
Age (years)	0.043	0.015	1.044	1.014	1.074	*0.004*	*0.046*
ISS	−0.034	0.023	0.967	0.924	1.012	0.144	<*0.001*
SBP (mmHg)	−0.018	0.011	0.982	0.961	1.003	0.092	<*0.001*
Temperature (°C)	−0.300	0.179	0.741	0.521	1.053	0.094	<*0.001*
Hb (mmol/L)	0.009	0.213	1.009	0.665	1.531	0.965	<*0.001*
Base excess (mmol/L)	−0.070	0.066	0.933	0.819	1.062	0.294	<*0.001*
Lactate (mmol/L)	0.047	0.100	1.048	0.863	1.274	0.635	<*0.001*
RBC/24 h (n)	0.011	0.026	1.011	0.960	1.065	0.673	<*0.001*
Noradrenaline (µg/kg/min)	2.148	0.534	8.571	3.007	24.433	*0.000*	<*0.001*
Time to surgery (hours)	−0.062	0.163	0.940	0.683	1.294	0.705	*0.010*
Anticoagulation	−1.698	1.053	0.183	0.023	1.442	0.107	0.078
AIS head >3	1.358	0.640	3.887	1.109	13.627	*0.034*	<*0.001*
Out-of-hospital airway management	0.535	0.807	1.708	0.351	8.301	0.507	<*0.001*
Total incidence of complications	−0.053	0.601	0.949	0.292	3.083	0.930	0.052

ISS; injury severity score, SBP; systolic blood pressure, Hb; hemoglobin, RBC; red blood cell transfusion, AIS; abbreviated injury severity, CI; confidence interval. Logistic regression including data from n = 515 patients with multiple trauma; Hosmer-Lemeshow goodness of fit test: p = 0.869; Nagelkerke’s R^2^: 0.642; model chi-square: 140.5, p < 0.001. Sensitivity 59.4% (19/32) correctly predicted descendents, Specificity 98.8% (477/483) correctly predicted survivors; italicized p values indicate statistical significance (p < 0.05).

**Table 6:** Predictors of 30-day mortality.

Parameter	Regression coefficient	Standard error	Odds radio	95% CI lower value	95% CI upper value	Multivariate p value	Univariate p value
Constant	−8.961	7.338	0.000			0.222	
Age (years)	0.030	0.016	1.030	0.999	1.062	0.056	*0.003*
BMI	0.110	0.053	1.117	1.007	1.238	*0.036*	*0.008*
ISS	0.113	0.027	1.120	1.062	1.180	*0.000*	<*0.001*
Admission SBP (mmHg)	−0.014	0.008	0.986	0.971	1.001	0.064	<*0.001*
Admission Temperature (°C)	−0.022	0.201	0.978	0.659	1.451	0.912	<*0.001*
Admission Hb (mmol/L)	0.136	0.196	1.146	0.780	1.684	0.488	<*0.001*
Admission Base excess (mmol(L)	−0.095	0.078	0.910	0.780	1.060	0.226	<*0.001*
Admission Lactate (mmol/L)	0.086	0.119	1.089	0.863	1.375	0.471	<*0.001*
RBC/24 h	−0.030	0.050	0.971	0.881	1.070	0.550	<*0.001*
Noradrenaline/24 h (µg/kg/min)	2.384	0.866	10.843	1.988	59.155	*0.006*	<*0.001*
Time to surgery (hours)	−0.272	0.170	0.762	0.546	1.063	0.109	*0.008*
ICU LOS (days)	−0.172	0.034	0.482	0.788	0.899	*0.000*	<*0.001*
Male gender	−0.392	0.538	0.676	0.236	1.939	0.467	*0.022*
ASA >2	0.658	0.680	1.931	0.509	7.319	0.333	*0.023*
Admission 8 am–7 pm	0.760	0.579	2.138	0.687	6.651	0.189	*0.047*
AIS head >3	1.822	0.477	6.186	2.427	15.770	*0.000*	<*0.001*
Out-of-hospital airway management	0.268	0.507	1.308	0.484	3.533	0.597	<0.001
Total incidence of complications	−0.731	0.607	0.481	0.147	1.582	0.228	0.084

BMI; body mass index, ISS; injury severity score, SBP; systolic blood pressure, Hb; hemoglobin, RBC; red blood cell transfusion, OR; operating room, ICU LOS; intensive care unit length of stay, ASA; American Society of Anesthesiologists classification, AIS; abbreviated injury severity, CI; confidence interval. Logistic regression including data from n = 514 patients with multiple trauma; Hosmer-Lemeshow goodness-of-fit test: p = 0.176; Nagelkerke’s R2: 0.744; model chi-square: 283.8, p < 0.001. Sensitivity 72.2% (56/76) correctly predicted descendents, Specificity 98.2% (429/437) correctly predicted survivors; italicized p values indicate statistical significance (p < 0.05).

### Airway management

Emergency airway management was performed 401 times in 383 patients (72.8%), with an overall complication rate of 13.2% (n = 53) of the procedures (Table 2). Among them, out-of-hospital airway management was performed in 239 patients (62.4%), of whom 224 patients (93.7%) underwent tracheal intubation. The out-of-hospital complication rate was 16.7% (n = 40) and included multiple intubation attempts (n = 19), esophageal intubations (n = 10, four of which remained undetected until hospital admission), deep main-stem bronchial intubations (n = 8, seven right-sided and one left-sided; four of them detected in the resuscitation room and another four by CT), and misplaced supraglottic airway devices (n = 3). Supraglottic airway devices were used in 16 patients (laryngeal tubes n = 8, laryngeal mask airways n = 6, and Combitubes™ n = 2, respectively) and four patients underwent bag valve mask ventilation. Reasons were inability of tracheal intubation (n = 11) or primary supraglottic airway device approach during vehicle extrication (n = 5). Of these, five patients still underwent a successful tracheal intubation before hospital admission. In nine cases, another EMS physician (helicopter EMS) successfully performed the airway management. There was no patient with an out-of-hospital surgical airway. In the resuscitation room, 67 patients (17.5%) underwent advanced airway management (complication rate: 13.4%; n = 9). Of these, 39 patients (58.2%) had been admitted to the resuscitation room with unsafe airways despite a formal indication for out-of-hospital advanced airway management according to guideline recommendations (e.g., GCS <9, admission respiratory distress, SaO_2_ <90%). Two patients required fiber optic intubation due to massive facial injuries. Another two patients required tracheotomy under local anesthesia for progressive upper airway obstruction, one of whom was temporarily ventilated with a laryngeal tube. In the operating room, 74 intubations (19.3%) were performed without complications, whereas in the ICU, 21 intubations (5.5%) were associated with a complication rate of 19.0% (n = 4). Airway management-related deaths occurred in two patients (0.5%) due to undetected out-of-hospital esophageal intubations.

Complication rates of airway management were similar in the early treatment phase (EMS and resuscitation room) compared with the later treatment phase (operating room and ICU) (14.4 vs. 9.5%; p = 0.217). In univariate analysis (including all variables of Table 1 with exception of the outcome parameters), airway complications were only significantly associated with ISS (p = 0.033). ROC curve analysis revealed an ISS cut off value 32.5 (sensitivity 0.569, specificity 0.754; Table 7), which was exceeded by 56.9% of patients with and by 24.6% of patients without complications (p < 0.001). The odds ratio for complication due to exceedance of the cut-off was 4.033 (95% CI: 2.231–7.292).

**Table 7:** Predictive parameters of mechanical complications of different invasive emergency procedures from univariate statistical analysis.

Parameter	p value	Odds ratio	Lower 95% CI	Upper 95% CI
*Airway management*				
ISS >32.5*	<*0.001*	4.033	2.231	7.292
*Chest tube placement*				
Out-of-hospital chest tube placement	*0.002*	3.650	1.551	8.590
Out-of-hospital tracheal intubation	*0.033*	2.938	1.053	8.191
*Central venous catheterization*				
ISS >33.5*	*0.005*	2.588	1.314	5.098
Out-of-hospital tracheal intubation	0.056	2.178	0.966	4.910
*Arterial catheter placement*				
Admission serum lactate >4.95 mmol/L	*0.001*	3.394	1.654	6.962
Admission base excess <−4.05 mmol/L	*0.008*	2.431	1.239	4.770

CI; confidence interval, ISS; injury severity score, EMS; emergency medical service. *Cut off values from receiver operating characteristic (ROC) curve analysis; italicized p values indicate statistical significance (p < 0.05).

### Chest tube placement

Chest tube placement (unilateral or bilateral insertion) was performed 196 times in 157 patients (29.8%), with a complication rate of 15.8% (n = 31) (Table 2). Complications included tube kinking (n = 11), and malfunction (n = 20)^19^. During the out-of-hospital EMS treatment, 35 (17.8%) chest tube placements were performed, of which 9 patients (25.7%) underwent needle-decompression prior to chest tube placement. The out-of-hospital complication rate was 22.8% (n = 8). In the resuscitation room, 88 (44.9%) chest tube placements were performed (complication rate: 18.2%, n = 16). Chest tubes placed in the out-of-hospital setting and the resuscitation room period required immediate repositioning in 8.9% (n = 11) of the cases after CT diagnosis. In the operating room, 26 (13.2%) chest tubes placements were performed (complication rate: 11.5%; n = 3) compared with 47 (24.0%) chest tube placements in the ICU (complication rate: 8.5%; n = 4). Chest tube placement in the early treatment phase (EMS and resuscitation room) tended to yield more complications compared with the later treatment phase (operating room and ICU) (19.5 vs. 9.8%; p = 0.066). Univariate statistical analysis revealed chest tube-related complications to be associated with out-of-hospital placement (OR 3.650; 95% CI: 1.551–8.590, p = 0.002) and out-of-hospital airway management (OR 2.938; 95% CI: 1.053–8.191, p = 0.033) (Table 7).

### Central venous catheterization

CVCs were placed 360 times in 318 patients (60.4%), with a complication rate of 11.4% (n = 41) (Table 2). Three patients (0.8%) received CVC placements during the out-of-hospital EMS treatment (one complication). During resuscitation room management, 191 (53.0%) CVC placements were performed, with a complication rate of 12.5% (n = 24). In the operating room, 47 (13.0%) CVCs were placed (complication rate: 6.4% (n = 3), and in the ICU, 119 (33.0%) CVCs were placed (complication rate: 10.9%; n = 13), respectively. The most common puncture sites were the subclavian veins (n = 277; 76.9%), followed by the femoral veins (n = 55; 15.3%) and internal jugular veins (n = 28; 7.8%), as described in our SOP. The mechanical complications were arterial puncture (n = 15), large hematomas (n = 13), failure (n = 6), arterial catheterization (n = 3) pneumothorax (n = 2), and guidewire loss (n = 2). One patient suffered guidewire loss during catheterization of the right femoral vein following unintended right subclavian artery catheterization, requiring angiographic intervention. Complication rates of the early treatment phase (EMS and resuscitation room) were comparable to the later treatment phase (operating room and ICU: 12.9 vs. 9.6%; p = 0.334). Univariate statistical analysis confirmed ISS >33.5 (OR 2.588; 95% CI: 1.314–5.098, p = 0.005) and out-of-hospital airway management (OR 2.178; 95% CI: 0.966–4.910) to be associated with CVC-related complications (Table 7).

### Arterial line placement

Arterial line placement was performed 434 times in 416 patients (79.1%) with a complication rate of 9.4% (n = 41) (Table 2). In this study, arterial puncture was not performed in the out-of-hospital setting. In the resuscitation room, 291 (67.0%) arterial line placements were performed (complication rate: 11.0%; n = 32) compared with 54 (12.4%) in the operating room (no complications) and 89 (20.5%) in the ICU (complication rate: 10.1%; n = 9). The preferred puncture sites were femoral arteries (n = 346; 79.7%) compared with radial arteries (n = 88; 20.3%). Mechanical complications were multiple punctures (n = 32; 78.0%), failed attempts (n = 6; 14.6%) and lost guidewires (n = 3; 7.3%). Univariate statistical analysis revealed arterial line placement-related complications to be solely associated with admission serum lactate >4.95 mmol/L (OR 3.394; 95% CI: 1.654–6.962, p = 0.001) and admission base excess <−4.05 mmol/L (OR 2.431 95% CI: 1.239–4.770, p = 0.008) (Table 7).

## Discussion

Our study revealed that mechanical complications of invasive emergency procedures occurred in 26.2% of severely injured patients. Most complications were recognized immediately or were detected using routine emergency CT diagnostic, whereas a considerable number of out-of-hospital complications were recognized at hospital admission. A main finding is that mechanical complications were associated with out-of-hospital airway management and ISS. However, the predictive power of the statistical model including these two parameters is rather restricted since it predicted only 16% of the variance of the residuals. We presume that other factors not included in our model (e.g., clinical experience under non-emergency conditions, mental status, and ability for fast decision making of the attending physicians) might be of considerable importance that should be addressed in future studies. Although some single comparisons showed significant differences using a univariate analysis, multivariate logistic regression analysis confirmed that time of day for the procedure, pre-injury morbidity, admission hypothermia, admission systolic blood pressure (SBP), noradrenaline dosage and numbers of packed red blood cells did not independently predict mechanical complications. This result most likely suggests an operator-dependent association with mechanical complications and a high complexity of the emergency setting. Patients suffering mechanical complications were associated with longer ICU LOS, which led to higher economic burden and risk of further ICU-related complications. Mortality tended to be higher but remained below statistical significance compared with patients without mechanical complications in univariate analysis. Further multivariate tests confirmed that the incidence of mechanical complications had no statistical effect on both 24 hour and 30 day mortality, as discussed later.

Out-of-hospital EMS and resuscitation room environments tended to be associated with more severe complications compared with the operating room and ICU, although remaining below significance levels. Particularly, out-of-hospital airway management, which was performed in almost half of our study collective, accounted for two procedure-related deaths due to undetected esophageal tube malposition. Tragically, capnography as a method of malposition detection was not used in both fatal cases. Furthermore, out-of-hospital airway management complications were associated with increased ICU LOS, which led to higher economic burden and risk of further ICU-related complications. During the study period, we observed a relatively high number of patients who presented to the resuscitation room without proper airway management, despite formal indications. The importance of appropriate advanced airway management in trauma patients has been demonstrated in numerous studies^1–3^. One study in patients with severe traumatic brain injury who underwent out-of-hospital rapid sequence intubation revealed an increased rate of favorable neurological outcome at 6 months compared with patients who were intubated in the hospital^12^. Furthermore, trauma patients, especially those presenting with impaired consciousness, are prone to accidental tracheal aspiration of stomach contents and blood, which possibly further impairs the outcome^20,21^. Therefore, first-pass intubation success plays a key role in airway safety during emergency care^22^. Bag valve mask ventilation and pharyngeal tubes may be helpful to provide oxygenation and to bridge the time until a definite airway can be established. The use of supraglottic airway devices are second-line approaches when tracheal intubation is not possible or are first-line approaches for providers without the necessary skills according to German recommendations^23^. For emergency airway management, numerous studies and guidelines have been developed and published^22–29^. The main aspects and key quality indicators are the development of difficult airway management algorithms and the implementation of capnography and videolaryngoscopy into standard clinical practice.

With regard to pleura decompression, guidelines suggest that minithoracotomy and chest tube placement should be established in response to a clinical suspicion of tension pneumothorax immediately following needle decompression^2,3^. We observed a relatively low rate of needle decompression prior to chest tube insertion in the out-of-hospital setting. This might reflect the physician-based EMS system suggesting a more aggressive approach to tension pneumothorax suspicion compared to paramedic settings. Chest tube misplacement and malfunction under emergency conditions are known issues in trauma care. Our overall complication rate of 15.8% confirms the data of previous studies ranging from 5 to 38%^6,30^. We observed an association of mechanical complications of chest tube placement with out-of-hospital insertion, which highlights the special risk constellation for this procedure under time-critical conditions and in hostile environments. The increasing availability of portable ultrasound devices may help to detect pneumothorax earlier and independently from uncertain clinical investigation and time-consuming chest radiography^31,32^.

Large-bore CVC placement for high-volume fluid resuscitation is a frequently performed procedure in severely injured patients^9,10,33–35^. CVC placement in the out-of-hospital EMS setting is uncommon and was performed very rarely in this study. However, other centers have reported success rates comparable to in-hospital emergency settings^7^. The occurrence of mechanical complications related to CVC placement in the resuscitation room was relatively high considering that only experienced anesthetists performed this measure. This may depend on the choice of vascular anatomy (jugular veins are usually not accessible due to cervical spine immobilization devices), landmark-guided puncture approaches and impaired venous filling due to poor clinical conditions^36^. Strategies to reduce CVC-related mechanical complications may be the consequent use of puncture guidance using vascular ultrasound, endovascular electrocardiography, catheterization of femoral veins, use of short introducer sheaths, and avoidance of CVCs when appropriate numbers and sizes of peripheral cannulas can be established.

Arterial line placement provided the lowest complication rate of all four invasive emergency procedures analyzed in our study. Although arterial catheterization is a frequently performed intervention in acute care, data among severely injured patients are scarce. The German S3-guideline for managing severely injured patients recommends that ventilation should be monitored using arterial blood gas analysis after hospital admission^2,3^. Common mechanical complications include multiple punctures and failure due to hypotension caused by hemorrhagic shock or circulatory effects of anesthetic substances, as well as peripheral arterial occlusion disease (and consecutive chronic intravascular calcification)^37^. Interestingly, we found an association between arterial line placement complication and high admission serum lactate and negative base excess in our patients, suggesting that vascular access was more difficult in patients with severe shock. Although ultrasound guidance for arterial line placement is neither common in trauma resuscitation nor was it used in our present study, it may increase success rates and reduce performance times and complication rates and thus should be recommended for safety reasons^38^.

Guidelines or national recommendations for emergency invasive vascular access (either CVC or arterial line placement) are currently not available. Invasive vascular access should consider different trauma resuscitation strategies depending on the hospital infrastructure (e.g., CT in or near the resuscitation room), availability of resources (e.g., sonography, blood gas analysis, and transfusion protocols), clinical presentation (e.g., shock and cardiopulmonary resuscitation), anatomy (e.g., vascular access sites and chest tube insertion) and aspects of time management (e.g., immediate, early or delayed surgery) and safety performance^36^.

We acknowledge the general limitations of retrospective studies. Due to a possible reporting bias, complication rates may be higher in prospective controlled studies when complications are assessed by an independent observer who is not involved in direct patient care. The training levels and medical professions of EMS physicians were not assessed in this study, which may have been different compared with in-hospital settings. Furthermore, mechanical complications in patients who did not survive in the out-of-hospital setting and thus did not reach the hospital could not be analyzed. On the other hand, we only included patients with ISS >15 which in fact poses a selection bias because patients ISS <16 who underwent invasive emergency procedures (and their complications) have not been accessed in this study. A future study design may include matching of patients of the entire ISS spectrum and other risk factors with respect to the incidence of mechanical complication. Another limitation of the study is that the patients were not randomized to vascular access sites, which may have introduced a study bias. In addition, we present data of a single center that should be interpreted with caution and may not be comparable with other settings.

Although this study was unable to answer the question of whether the mechanical complications of emergency procedures are inevitable, a considerable proportion of observed complications might have been avoidable by the consequent application of available and recommended technologies (e.g., capnography, video laryngoscopy and sonography).

Regarding their relevance for the incidence of mechanical complications, both high ISS and need for out-of-hospital airway management (resulting subsequently in mechanical ventilation in the majority of cases) implicate together an increased impairment of patient condition. Both intensified the number of required emergency procedures for diagnostics and treatment, and it was most likely the complexity of the patient presentation in the acute care setting which enhanced the number of complications, but not vice versa.

Although mechanical complications were associated with increased morbidity they did not significantly contribute to mortality in our study. Nevertheless, the necessity of each procedure should be reflected critically in a case-by-case decision. Moreover, it should be kept in mind that any mechanical complication provides the potential for further harm.

## Methods

### Study design and setting

Prospectively collected data of consecutive patients with severe and multiple injuries who underwent invasive emergency procedures with or without mechanical complications were reviewed. The study was conducted at the Leipzig University Hospital. Data collection involved patients treated between January 2010 and December 2015. Ethical approval for the study was obtained from the institutional review board of the Leipzig Faculty of Medicine (No. 137-15-20042015). The study was registered retrospectively at www.researchregistry.com under the unique identifying number (UIN) researchregistry2880 (August 13, 2017).

### Eligibility criteria

Inclusion criteria were age >14 years, admission directly from the scene of the accident, and injury severity score (ISS) >15.

### Definitions

The main outcome measure was the occurrence of mechanical complications classified as multiple intubation attempts; unsuccessful tracheal intubation requiring supraglottic airway devices or bag valve mask ventilation for rescue management; undetected esophageal and bronchial intubation at resuscitation room admission; chest tube malfunction; vascular access with multiple puncture attempts, accidental arterial puncture, or associated hematoma and/or pneumothorax; CVC and arterial line malpositions; and loss of guidewires.

Definitions of risk factors for potential complications included age, gender, pre-injury morbidity (body mass index (BMI) >29 and <21, American Society of Anesthesiologists (ASA) classification >2, or pre-injury anticoagulation medication), admission systolic blood pressure (SBP) <90 mmHg, admission hypothermia <35.2 °C, admission hemoglobin (Hb), admission base excess (BE), admission serum lactate, ISS, abbreviated injury severity (AIS) head >3, environment of the performed procedures (EMS, resuscitation room, operating room, or ICU), number of red blood cell (RBC) transfusions, noradrenaline dosage.

Secondary endpoints were ventilator days, ICU length of stay (LOS), 24-hour mortality and 30 day mortality.

### General management

In Germany, out-of-hospital emergency physicians are part of an EMS team and perform on-scene trauma resuscitation^2,3^. At our center, resuscitation room management is organized according to the recommendations of the German Society of Trauma Surgery using an interdisciplinary trauma team and a standardized advanced trauma life support (ATLS®) approach. Depending on the trauma mechanism, clinical presentation and point-of-care blood gas analysis, severely injured patients are either scheduled for whole body CT (located in direct proximity to the resuscitation room) or for immediate surgery. The attending anesthesia team (consultant and resident) is responsible for airway management, ventilation, fluid resuscitation, coagulation and transfusion management. According to the resuscitation room standard operating procedure (SOP) of our center, cannulation of the right subclavian vein (20 cm, 12 F Shaldon catheter) and the left femoral artery (Seldinger-wire system) is primarily recommended for emergency vascular access in severely injured patients. The surgical team is responsible for clinical examination, mechanical bleeding control (e.g., pelvic clamp or tourniquet), tube thoracostomy, and extended focused assessment sonography for trauma (eFAST).

### Statistical analysis

Data are reported as the mean ± standard deviation and numbers (percentage). Statistical comparisons between patients with and without complications were performed using the χ^2^ test for qualitative data. Student’s *t* test was applied for the analyses of quantitative data. When the Levene test for the homogeneity failed, the Mann-Whitney U-test was used. The alpha level of significance was set at 0.05. All tests were two-sided. A primary univariate logistic regression analysis was performed to identify independent predictors of procedure complications. Risk factors with p values of <0.1 were included in the multivariate model to identify significant independent risk factors. In order to characterize general statistics of the logistic regression we report the model chi-square, its p value (expected to be <0.05), Nagelkerke’s R^2^ (effect size measure, analogous to the square of a regression coefficient), the results of the Hosmer-Lemeshow goodness of fit statistics (expected to be >0.05), and the sensitivity of the statistical model within the investigated patient sample. With two further exploratory logistic regression analysis we aimed at identifying the confounders of 24 hour-mortality and of 30 day-mortality. Multiple regression was used to determine covariates of ICU length of stay. This variable was not normally distributed and we included all potential risk factors with univariate p < 0.1 in Spearman’s rank correlation analysis or in the Mann-Whitney-U test, respectively. Non-linearity of the residuals, i.e. lack of autocorrelation in the model was checked with the Darbin-Watson test revealing a value near 2. Due to heteroscedasticy (heterogeneity of variances) among the residuals a robust model was applied in which confidence intervals and standard errors of the regression coefficients were based on 1,000 bootstrap samples. The correlation coefficient R is given as effect size measure. Receiver operating characteristics (ROC) analysis was used to investigate the relationship between predictors and the incidence of mechanical complications. ROC-curves were used in order to display the predictive value by plotting the true-positive rate (sensitivity) against the false-positive rate (1-specificity) at various threshold settings. The area under the curve (AUC) was compared to the area under the diagonal line of identity which corresponds to random chance (i.e. true positive rate equals false positive rate). In order to identify the corresponding numeric result for an optimal cut-off point, the Youden-index (J) was calculated. ROC analysis was used to define cut off values for univariate significant numerical predictors which were prior separately determined for the different types of complications concerning airway management, chest tube placement, CVC placement and arterial line placement, respectively. Following a chi-square test, we calculated for each cut off value the odds ratio for the increased incidence in the complication rate together with its 95% confidence interval. Computations were performed using SPSS 24.0, IBM Corp. Armonk, NY, USA.

### Abbreviations

AIS: Abbreviated injury score; ASA: American Society of Anesthesiologists; BE: Base excess; BMI: Body mass index; CI: Confidence interval; CT: Computed tomography; CVC: Central venous catheter; ED: Emergency department; EMS: Emergency medical service; Hb: Hemoglobin; ICU: Intensive care unit; ISS: Injury severity score; LOS: Length of stay; OR: Odds ratio; RBC: Red blood cell; SBP: Systolic blood pressure; SOP: Standard operating procedure.

### Ethics approval and consent to participate

Ethical approval was obtained from the IRB of the Leipzig Faculty of Medicine. Consent to participate was not applicable owing to the retrospective nature of the study.

### Availability of data and materials

The datasets used and/or analyzed during the current study are available from the corresponding author upon reasonable request.

## References

[1] McCullough AL, Haycock JC, Forward DP, Moran CG (2014). Early management of the severely injured major trauma patient. Br. J. Anaesth..

[2] AWMF S3-Leitlinie Polytrauma/Schwerverletzten-Behandlung, Registrierungsnummer 012–019, AWMF, Düsseldorf, http://www.awmf.org/leitlinien. AccessedAugust 24, 2017.

[3] Hilbert-Carius P (2017). [Care for severely injured persons: Update of the 2016 S3 guideline for the treatment of polytrauma and the severely injured]. Anaesthesist.

[4] Lockey D, Crewdson K, Weaver A, Davies G (2014). Observational study of the success rates of intubation and failed intubation airway rescue techniques in 7256 attempted intubations of trauma patients by pre-hospital physicians. Br. J. Anaesth..

[5] Rognås L, Hansen TM, Kirkegaard H, Tønnesen E (2013). Pre-hospital advanced airway management by experienced anaesthesiologists: a prospective descriptive study. Scand. J. Trauma Resusc. Emerg. Med..

[6] Maybauer MO, Geisser W, Wolff H, Maybauer DM (2012). Incidence and outcome of tube thoracostomy positioning in trauma patients. Prehosp. Emerg. Care.

[7] Fyntanidou B (2009). The use of central venous catheters during emergency prehospital care: a 2-year experience. Eur. J. Emerg. Med..

[8] Fevang E, Perkins Z, Lockey D, Jeppesen E, Lossius HM (2017). A systematic review and meta-analysis comparing mortality in pre-hospital tracheal intubation to emergency department intubation in trauma patients. Crit. Care.

[9] Choron RL, Wang A, Van Orden K, Capano-Wehrle L, Seamon MJ (2015). Emergency central venous catheterization during trauma resuscitation: a safety analysis by site. Am. Surg..

[10] Scalea TM (1994). Percutaneous central venous access for resuscitation in trauma. Acad. Emerg. Med..

[11] Green RS, MacIntyre JK (2009). Critical care in the emergency department: an assessment of the length of stay and invasive procedures performed on critically ill ED patients. Scand. J. Trauma Resusc. Emerg. Med..

[12] Bernard SA (2010). Prehospital rapid sequence intubation improves functional outcome for patients with severe traumatic brain injury: a randomized controlled trial. Ann. Surg..

[13] Sobrino J, Shafi S (2013). Timing and causes of death after injuries. Proc. (Bayl. Univ. Med. Cent)..

[14] Ondruschka B (2017). Additional emergency medical measures in trauma-associated cardiac arrest. Anaesthesist.

[15] Kleber C, Giesecke MT, Tsokos M, Haas NP, Buschmann CT (2013). Trauma-related preventable deaths in Berlin 2010: need to change prehospital management strategies and trauma management education. World J. Surg..

[16] Davis JS (2014). An analysis of prehospital deaths: Who can we save?. J. Trauma Acute Care Surg..

[17] Roy N (2017). Learning from 2523 trauma deaths in India- opportunities to prevent in-hospital deaths. BMC Health Serv. Res..

[18] Lotan E, Portnoy O, Konen E, Simon D, Guranda L (2015). The role of early postmortem CT in the evaluation of support-line misplacement in patients with severe trauma. AJR Am. J. Roentgenol..

[19] Struck MF, Leclair N, Wrigge H (2015). Bilateral false-positive tube thoracostomy in helicopter emergency medical service. Air Med. J..

[20] Radu, R. R. *et al*. Prevalence and in-hospital outcome of aspiration in out-of-hospital intubated trauma patients. *Eur. J. Emerg. Med*. 10.1097/MEJ.0000000000000465. [Epub ahead of print] (2017 Mar 31). 10.1097/MEJ.0000000000000465 28368907

[21] Fawcett VJ (2015). Pre-hospital aspiration is associated with increased pulmonary complications. Surg. Infect. (Larchmt).

[22] Bernhard M, Becker TK, Gries A, Knapp J, Wenzel V (2015). The First Shot Is Often the Best Shot: First-Pass Intubation Success in Emergency Airway Management. Anesth. Analg..

[23] Piepho T (2015). S1 guidelines on airway management: Guideline of the German Society of Anesthesiology and Intensive Care Medicine. Anaesthesist.

[24] Tobin JM (2013). A checklist for trauma and emergency anesthesia. Anesth. Analg..

[25] Hossfeld B (2016). Recommended practice for out-of-hospital emergency anaesthesia in adults: Statement from the Out-of-Hospital Emergency Anaesthesia Working Group of the Emergency Medicine Research Group of the German Society of Anaesthesiology and Intensive Care. Eur. J. Anaesthesiol..

[26] Lockey DJ (2015). Advanced airway management is necessary in prehospital trauma patients. Br. J. Anaesth..

[27] Helm M, Schuster R, Hauke J, Lampl L (2003). Tight control of prehospital ventilation by capnography in major trauma victims. Br. J. Anaesth..

[28] Silvestri S (2017). Endotracheal tube placement confirmation: 100% sensitivity and specificity with sustained four-phase capnographic waveforms in a cadaveric experimental model. Resuscitation.

[29] Lewis SR, Butler AR, Parker J, Cook TM, Smith AF (2016). Videolaryngoscopy versus direct laryngoscopy for adult patients requiring tracheal intubation. Cochrane Database Syst. Rev..

[30] Kong VY, Oosthuizen GV, Sartorius B, Keene C, Clarke DL (2014). An audit of the complications of intercostal chest drain insertion in a high volume trauma service in South Africa. Ann. R. Coll. Surg. Engl..

[31] Sauter TC, Hoess S, Lehmann B, Exadaktylos AK, Haider DG (2017). Detection of pneumothoraces in patients with multiple blunt trauma: use and limitations of eFAST. Emerg. Med. J..

[32] Gaydos S (2012). Clinical auscultation in noisy environments. J. Emerg. Med..

[33] Pappas P, Brathwaite CE, Ross SE (1992). Emergency central venous catheterization during resuscitation of trauma patients. Am. Surg..

[34] Ives C (2012). Ten years of mechanical complications of central venous catheterization in trauma patients. Am. Surg..

[35] Odendaal J (2017). Mechanical complications of central venous catheterisation in trauma patients. Ann. R. Coll. Surg. Engl..

[36] Struck MF (2017). Anaesthesia procedures and invasive vascular access in severely injured patients at trauma room admission in Germany: An online survey. Anaesthesist.

[37] Salmon AA (2010). Analysis of major complications associated with arterial catheterisation. Qual. Saf. Health Care.

[38] Zochios VA, Wilkinson J, Dasgupta K (2014). The role of ultrasound as an adjunct to arterial catheterization in critically ill surgical and intensive care unit patients. J. Vasc. Access.

